# Immunologic Regulation in Pregnancy: From Mechanism to Therapeutic Strategy for Immunomodulation

**DOI:** 10.1155/2012/258391

**Published:** 2011-11-03

**Authors:** Shyi-Jou Chen, Yung-Liang Liu, Huey-Kang Sytwu

**Affiliations:** ^1^Department of Pediatrics, Tri-Service General Hospital, National Defense Medical Center, Taipei 114, Taiwan; ^2^Department of Microbiology and Immunology, National Defense Medical Center, Taipei 114, Taiwan; ^3^Department of Obstetrics and Gynecology, Tri-Service General Hospital, National Defense Medical Center, Taipei 114, Taiwan; ^4^Graduate Institute of Medical Sciences, National Defense Medical Center, Taipei 114, Taiwan; ^5^Graduate Institute of Life Sciences, National Defense Medical Center, Taipei 114, Taiwan

## Abstract

The immunologic interaction between the fetus and the mother is a paradoxical communication that is regulated by fetal antigen presentation and/or by recognition of and reaction to these antigens by the maternal immune system. There have been significant advances in understanding of abnormalities in the maternal-fetal immunologic relationship in the placental bed that can lead to pregnancy disorders. Moreover, immunologic recognition of pregnancy is vital for the maintenance of gestation, and inadequate recognition of fetal antigens may cause abortion. In this paper, we illustrate the complex immunologic aspects of human reproduction in terms of the role of human leukocyte antigen (HLA), immune cells, cytokines and chemokines, and the balance of immunity in pregnancy. In addition, we review the immunologic processes of human reproduction and the current immunologic therapeutic strategies for pathological disorders of pregnancy.

## 1. Introduction

In 1953, Medawar first proposed the concept of immune tolerance, giving as an example the case of the fetal allograft [1, 2]. He addressed the hypothesis that the semiallogeneic fetus is able to survive because the immunologic interaction between mother and fetus is regulated and inhibited, and that this is because of either a lack of fetal antigen expression resulting from the anatomic separation of the mother from the fetus or a functional suppression of maternal lymphocytes. The exact mechanisms required to induce immunologic tolerance of the fetus are not entirely understood. Despite close contact between fetal trophoblasts and maternal immune cells, there is a lack of antigen stimulation of maternal lymphocytes [3–5].

Successful pregnancy has been considered a biologic example of semiallogeneic graft acceptance, in which the semiallogeneic fetus is protected from immune attack from the mother. Interestingly, the so-called semiallogeneic conceptus actually consists of the trophoblast cells at the maternal-conceptus interface [6]. During the first trimester of human pregnancy, the placenta develops into a dividing villous formation with differentiation of characteristic trophoblast cell types having different functions. Cytotrophoblast progenitors found in the villi follow two differentiation pathways: some fuse to form the multinucleated syncytiotrophoblast layer that encases the floating villi of the placenta, providing the interface with maternal blood to regulate oxygen and protein transport [7], while others follow an invasive pathway and differentiate into extravillous trophoblast (EVT) cells [8]. These cells migrate from the villous tips in columns that anchor the placenta to the maternal decidua, and EVT cells form the cytotrophoblast shield over the decidua as well as migrating and invading the decidua. Invasive EVT cells play an active role in the remodeling processes that occur in the uterine spiral arteries [9]. In human hemochorial placenta, fetal trophoblast cells appear to be in extremely close contact with the maternal immune cells, based on the observation that EVT cells invade the maternal decidua. Thus, immunologic interrelations between mother and fetus during pregnancy are thought to occur in the decidua [10, 11]. The invasive competence of EVT cells is most dominant during the first trimester and declines afterward.

The major cellular component of the decidua is decidual stromal cells (DSCs). DSCs exert different immune activities that have emerged as relevant to the immunologic interaction between mother and fetus and may lead to either a normal pregnancy or abortion [12, 13]. Recent attention has focused on current knowledge of the effects of pregnancy on the immune response, both peripherally and in the decidua, leading to a discussion on fetal mechanisms for escaping maternal immune attack and the development of immunomodulatory therapeutic strategies for reproductive problems [14]. Understanding the immunologic processes that occur in normal conception will significantly improve our awareness of pathological conditions and suggest strategies to manage associated human reproductive disorders such as abortion, preeclampsia, and preterm delivery [15].

## 2. Alteration of Human Leukocyte Antigen Expression during Pregnancy

Human leukocyte antigens (HLAs) are also called “transplantation” antigens because they comprise the most powerful stimulators of graft rejection. However, novel HLAs expressed in the fetal membranes are tolerogenic rather than immunogenic [16, 17], and although anti-paternal HLA antibodies are common in pregnant women, they do no damage [18]. One of the fundamental and absorbing paradoxes of life is the immunologic tolerance during reproduction involving the survival and symbiosis of the genetically distinct fetus and its mother [5]. Thus, the mechanisms underlying maternal tolerance are usually effective and raise the essential issue of how immune privilege might be established under the natural conditions of pregnancy to guarantee viability of the embryo/fetus [19–21].

The expression of major histocompatibility complex (MHC) proteins at the interface between mother and fetus is tightly controlled in mammalian pregnancy [22]. The expressed MHC class I genes are subdivided into class Ia, which includes *HLA-A*, -*B*, and -*C* and class Ib, which includes *HLA-E*, -*F*, and -*G*. HLA class II (*HLA-D*) genes are not translated in human trophoblast cells even under inducing conditions where they are transcribed [23, 24] and MHC class II is also undetected in both villous and extra-villous human trophoblast cells [25]. Human trophoblast cells express one MHC class Ia molecule (HLA-C) and all three class Ib molecules. In human placenta, the cell-surface expression of MHC class I molecules by the fetal trophoblast is limited to loci of low polymorphism, *HLA-G*, *HLA-E*, and *HLA-C* [26, 27], but the fetal trophoblast cells do not express the MHC class Ia antigens HLA-A and HLA-B [19, 28] that are responsible for the rapid rejection of allografts in humans. The *HLA-C* gene is moderately polymorphic and could possibly stimulate maternal antifetal acquired immunity if the paternal alleles differ from the maternal. Interactions between HLA-C and decidual natural killer (NK) cells may also facilitate trophoblast invasion into maternal tissue: Tilburgs et al. demonstrated that pregnancies with an HLA-C-mismatched child induce an increased percentage of activated T cells in decidual tissue. In addition, HLA-C-mismatched pregnancies exhibit a decidual lymphocyte response to fetal cells and contain functional regulatory T cells in decidual tissue, whereas HLA-C-matched pregnancies do not. This suggests that in uncomplicated pregnancies, decidual T cells exclusively recognize fetal HLA-C at the maternal-fetal boundary, but are prevented from inducing a destructive immune response [29]. Despite this, allelic differences at the HLA-C locus do not seem to be a contributory factor in infertility or termination of pregnancy.

HLA-G was the first of the HLA class Ib molecules expressed by trophoblast cells to be identified and remains an antigen of great interest and a focus of experimental evaluation [19, 20, 30]. Understanding the molecular and biochemical features of the *HLA-G *gene and its products may improve our ability to determine the ways in which HLA-G can affect pregnancy [31]. Multiple reports indicate that levels of HLA-G may predicate reproductive success [20, 32, 33]. As a consequence, fertility physicians are anxious to identify commercially available enzyme-linked immunosorbent assays or other assays that will accurately report levels of HLA-G in the blood of patients with suboptimal fertility [31].

Although it has been proposed that HLA-G may be an evolutionary artifact without function [34], recent studies using HLA-G proteins from transfected cells indicate that these proteins may regulate immune cells and thus may be integral to immune privilege in pregnancy [20, 35]. HLA-G proteins probably target all of the major immune cell subsets [33, 35, 36]. Moreover, Blanco et al. showed that the expression of HLA-G by DSC preserved their potential to control the cytotoxic activity of NK cells against trophoblast and the physiological decay (by apoptosis) of DSC [15].

In the following, we discuss further the complex immune interactions of human reproduction. We describe the activities in pregnancy of lymphocytes, NK cells, uterine (u)NK cells, chemokines, and antigen-presenting cells (APCs) including macrophages.

## 3. Trophoblast Cells and Immunologic Balance during Pregnancy

An essential issue in pregnancy is how the fetal-placental unit escapes maternal immune rejection. Although fetal and maternal cells interact throughout pregnancy, the fetus naturally continues as a privileged site not subject to rejection [37, 38]. In most of the species that have been studied, expression of MHC molecules by trophoblast cells is repressed, apparently as a strategy to avoid recognition and destruction by the maternal immune system. In recent years, studies of equine pregnancy have advanced the field of reproductive immunology [39]. The trophoblast cells of the horse are unique in the combination of spatial and temporal regulation of MHC expression that they exhibit during placentation. The allantochorion trophoblasts, which comprise the majority of the maternal-fetal interface, do not express MHC class I proteins, although some mRNA can be detected in these cells [40]. During a short window in early pregnancy, the trophoblasts of the chorionic girdle and endometrial cups transiently express very high levels of polymorphic MHC class I antigens of both maternal and paternal origin [41]. The transcription of both polymorphic and nonpolymorphic MHC class I loci in invasive trophoblasts and the high levels of cell-surface expression of the polymorphic antigens set apart the equine model. Maternal and paternal MHC antigens are both expressed on horse trophoblast, and the mare frequently produces cytotoxic antibodies to the paternal alloantigen shortly after chorionic girdle trophoblast invasion [41]. The expression of HLA-G and HLA-E antigens by the trophoblast may also inhibit cytolysis by natural killer cells [42, 43]. Bacon et al. further demonstrated that, in the equine model, functional alloantigen presentation by the trophoblast can be a normal part of early pregnancy [40].

Peripheral blood lymphocytes isolated from pregnant mares demonstrate a reduced capacity to develop into effector cytotoxic T lymphocytes capable of lysing target cells from the breeding stallion [44]. This reduction in T-cell-mediated alloreactivity reverts after parturition or pregnancy termination and is not observed in males or nonpregnant females. Work by several groups has demonstrated trophoblast-produced soluble factors that may create such an environment by modulating the proliferation and blastogenesis of maternal lymphocytes. Extracts from day 80 placenta have been shown to inhibit the proliferation of maternal lymphocytes, and coculture of chorionic girdle trophoblasts with maternal lymphocytes caused a decrease in proliferation and a reduction in cytokine production [45, 46]. In conclusion, the pregnant mare's immune responses to the trophoblast of her developing placenta are fascinating in their complexity. By providing a window into the nature of maternal-fetal interactions, the horse has illuminated immunologic events not easily detectable in other species.

It is well recognized that there is intimate contact between maternal tissue and the EVT cells that invade the decidua, and that there are high numbers of different types of leukocytes present within the stromal compartment of the luteal-phase endometrium, which increase in first-trimester decidua [10, 47]. Human decidua contain abundant immune cells during gestation, with more than 30% of stromal cells in first-trimester decidua expressing the leukocyte common antigen CD45 [10]. There are four major populations of decidua leucocytes present in early pregnancy: uNK cells, macrophages, dendritic cells (DC), and T cells [48]. Among these, the most abundant are uNK cells (CD56^+^CD16^−^ NK cells), macrophages (CD38^+^CD2^±^CD3^−^CD16^−^CD68^+^), and CD3^+^ T cells (CD8^+^ and rare CD4^+^), while B cells are virtually undetectable [11, 49, 50]. The increase in leukocyte numbers during the initial stage of pregnancy suggests that influx and/or proliferation of decidual leukocytes is under endocrine influence. Intimate contact between EVT cells and decidual leukocytes has been demonstrated by electron microscopy and immunohistochemistry, suggesting that there are paracrine interactions between maternal leukocytes and fetal cells [11, 51].

## 4. NK Cells and uNK Cells in Human Reproduction

In contrast with T and B cells, NK cells do not express somatically rearranged antigen-specific receptors [52]. The functions of NK cells are cell lysis and cytokine production, with individual cells having single or dual competence. For example, lysis is directed against virally infected cells and tumor cells. Interferon (IFN)-*γ*, which restricts viral infection, is a key cytokine product [53, 54]. The number of peripheral NK cells is decreased in pregnant women compared with nonpregnant women [55, 56], as is IFN-*γ* production [14].

uNK cells are essentially activated cytokine-producing NK cells [57] that share many characteristics with NK cells. Most peripheral NK cells express the surface marker CD16, an immunoglobulin (Ig) receptor, and have low expression of CD56, an adhesion molecule. In contrast, about 1% of peripheral lymphocytes are CD16^−^CD56^bright^ NK cells, and these CD16^+^CD56^+^ NK cells express high amounts of the vascular addressin L-selectin [58]. However, in humans, most uNK cells are CD56^bright^, but lack CD16 and L-selectin [59]. In women, uNK cells differentiate during every menstrual cycle, 3–5 days after the luteinizing hormone surge [60, 61]. The uNK cells may regulate trophoblast invasion into the decidua, myometrium, and uterine spiral arteries [62]. Postmitotic uNK cells are widely distributed within the decidua basalis, commonly (more than one quarter) associated intramurally and intraluminally with spiral arteries. From mid gestation, the number of uNK cells decreases. It appears that during the first half of gestation, uNK cells contribute to and sustain important changes in the maternal placental bed [54] by producing various soluble products including the angiogenic cytokines angiopoietin-2 and vascular endothelial growth factor [62]. In summary, uNK cells are appropriate residents of the maternal-fetal interface because of their unique function in supporting the adaptation of the blood vessels of the pregnant uterus [63].

## 5. Macrophages in Pregnancy

Macrophages present within the decidual immune cells during pregnancy have the potential to regulate divergent demands: maintenance of immune tolerance toward allogeneic fetal antigens and defense against the constant risk of infection by ascending and blood-borne pathogens [64]. The functional maturation of macrophages has been revisualized in a manner analogous to the well-supported concept of T helper (Th)1/Th2 polarization of effector T cells, by subcategorizing macrophage effector phenotypes as either M1 or M2 [65, 66]. Macrophages activated under the influence of proinflammatory cytokines and lipopolysaccharide are categorized as M1 type, secrete tumor necrosis factor (TNF) and interleukin (IL)-12, and participate in the progression of inflammation. In contrast, M2 macrophages are polarized by exposure to a milieu containing Th2 cytokines (IL-4, IL-10, and IL-13) and glucocorticoids [67]. M2 polarization is characterized by enhanced expression of innate immunity receptors, including scavenger receptors and the macrophage mannose receptor, as well as an upregulation of arginase activity, which counteracts nitric oxide synthesis [68]. In addition, M2 macrophages exhibit increased secretion of IL-1 receptor antagonist [69] and a reduction in IL-12 production that contributes to the functions of M2 macrophages in tissue repair and anti-inflammation [67]. The M2 polarization of decidual macrophages isolated from normal pregnancies indicates that their immunosuppressive activities are required for the maintenance of immunologic homeostasis during pregnancy. Simultaneously, recognition of hazardous microbes via toll-like receptors (TLRs) and C-type lectin receptors (CLRs) on macrophages is an essential mechanism for host defense in the decidua. Houser et al. propose two distinct subsets of CD14^+^ decidual macrophages in first-trimester decidual tissue, CD11c^HI^, and CD11c^LO^, which do not fit the conventional M1/M2 categorization [70]. CD11c^HI^ decidual macrophages express genes associated with lipid metabolism and inflammation, whereas CD11c^LO^ decidual macrophages express genes associated with extracellular matrix formation, muscle regulation, and tissue growth. The CD11c^HI^ decidual macrophages also differ from CD11c^LO^ decidual macrophages in their ability to process protein antigens and are likely to be the major APCs in the decidua. Moreover, these populations each secrete both proinflammatory and anti-inflammatory cytokines that may contribute to the balance that establishes maternal-fetal tolerance [70].

The M2 polarization of decidual macrophages isolated from normal pregnancies indicates that their immunosuppressive activities are required for the maintenance of immunologic homeostasis during pregnancy, while the recognition of dangerous microbes via TLRs and CLRs on macrophages is a key mechanism for host defense in the decidua. The remarkable phenotypic plasticity of uterine macrophages allows a balance of these seemingly discrepant activities, and defects in uterine macrophage function are closely linked to the pathophysiology of abnormal gestations, including those complicated by preeclampsia and preterm delivery [64].

## 6. Immune Tolerance and the Th Milieu during Human Reproduction

A successful pregnancy is the consequence of numerous complex interactions between the receptive uterus and the mature blastocyst under immunohormonal control [71, 72]. The Th1/Th2 ratio reaches a peak in the proliferative endometrium, significantly declines during the secretory phase and is at its lowest level in the early pregnancy decidua [73]. During the early phase of pregnancy, a successful implantation occurs in a proinflammatory microenvironment, and a Th1-type response is followed by a shift to Th2 to control endocrine and immune interactions [74–76]. Several cytokines such as TNF-*α* and IL-1 induce leukemia inhibitory factor expression in the stroma and epithelial cells, and through their receptors provide paracrine signals to both embryonic tissues and uterine epithelium during implantation [77]. Th1 responses may be suppressed during human pregnancy via downregulation of nuclear factor (NF)-*κ*B and T-bet transcription [78]. In addition, progesterone stimulates a Th2-type response, reduces inflammatory cytokines, and represses (potentially deleterious) allogeneic responses, conferring fetal survival [79, 80].

Decidual CD4^+^CD25^+^ T regulatory cells (Tregs) constitute about 14% of the total decidual CD4^+^ T cells and express glucocorticoid TNF receptor-related protein, OX40, and cytotoxic T lymphocyte antigen (CTLA)-4 [81]. CTLA-4 expression on Tregs may augment indoleamine 2,3-dioxygenase (IDO) expression by decidual and peripheral blood DCs and monocytes [82]. IDO is involved in maternal tolerance of the fetus by restraining the availability of tryptophan to T cells *in situ* in the uterine microenvironment [83]. Human pregnancy also involves expression of L-arginase, which exhausts arginine in the fetal-placental microenvironment, thus limiting maternal T-cell activity [84].

While uNK cells, macrophages, and DCs aid in orchestrating the balance between pro- and anti-inflammatory milieus over the course of gestation in humans, human decidua has also recently been shown to contain a small population of immature myeloid DCs [85]. Tregs in the uterus are thought to be mainly immunosuppressive. Decidual CD14^+^ cells express HLA-DR, but low levels of the costimulatory molecules CD80/CD86, suggesting that they could induce Tregs. The current recognized hypothesis predicts that the potential of trophoblastic antigens to induce a natural and tolerogenic maternal response engages Tregs, cytokines, chemokines, IDO, and galectin-1 derived from the fetoplacental unit [86–88], which suggests a possible strategy to treat pathological pregnancy via immunoregulation.

## 7. The Role of Chemokines in Successful Pregnancy

Chemokines are another important component that are involved in the complex immune network of the fetoplacental unit by adapting normal T cell trafficking and modulating the inflammatory process [89, 90]. We highlight CCL5 (also known as regulated upon activation, normal T cell expressed, and secreted (RANTES)), a proinflammatory chemokine that plays a part in the Th1 response, contributing to a tolerogenic response at immune-privileged sites in murine models, and which might function as an essential modulator of alloantigen-specific T-cell responses during normal pregnancy [91, 92]. Successful pregnancy is accompanied by an increase in RANTES serum levels, whereas these were found to be diminished in patients with recurrent spontaneous abortions [93]. In addition, Ramhorst et al. demonstrated, after treating Ishikawa cells—a human endocervical cell line—with recombinant RANTES and CCR5 (a receptor for RANTES), that there was a decrease in mRNA for CXCR4 (a chemokine receptor associated with a Th2 response) that correlated with an increase in expression of T-bet (the main transcription factor involved in development of a Th1 response) [94]. They also demonstrated that RANTES specifically suppresses alloactivated maternal T cells [95]. Thus, the high levels of progesterone present during normal human pregnancy, particularly at the maternal-fetal interface, would be predicted to promote RANTES production to levels required for the local induction of a tolerogenic immune response. This would suggest that RANTES may play an important role during maternal-fetal crosstalk, allowing trophoblast cell survival and a maternal tolerogenic response [96].

Evidence obtained either *in vitro* or *in vivo* has shown that three chemokine receptors, structurally related to signaling receptors, but incapable of activating signal transduction, the Duffy Antigen Receptor for Chemokines (DARC), D6, and CCX CKR, act as chemokine decoy receptors [97]. The best-known chemokine decoy receptor is the D6 molecule, a seven-transmembrane domain protein that shares 30%–35% sequence identity with signaling chemokine receptors, but cannot induce known chemokine receptor-signaling functions such as chemotaxis [98, 99]. D6 recognizes the majority of inflammatory CC chemokines and targets them for degradation [99]. D6 is strongly expressed by invading trophoblast cells and on the apical surface of syncytiotrophoblast cells [100]. Interestingly, Wessels et al. demonstrated that D6 is expressed in endometrial epithelium, uterine glands, and trophoblast; furthermore, in a model of spontaneous fetal loss in swine, a marked loss of D6 immunoreactivity was observed in arresting versus viable littermate attachment sites [101]. These results suggest that the absence of the scavenging function of D6 results in increased susceptibility to inflammation-driven fetal loss [102].

## 8. Immunomodulatory Molecules in Threatened Pregnancy

During pregnancy, the maternal immune system is obviously active and, under certain conditions, may contribute to fetal damage/death. Well-defined pathological processes include destruction of fetal erythrocytes (Rh antigen, erythroblastosis) and platelets (Human platelet alloantigens (HPA)-1 and HPA-2, alloimmune thrombocytopenia) by maternal antibodies and infections during pregnancy, where activated macrophages secreting high levels of Th1-type cytokines alter the fragile cytokine balance at the maternal-fetal interface [20, 103]. Takeshita et al. found that adipsin immunoreactivity was detected either at the decidua basalis in normal placentas or at the placental maze in absorbed placentas [104]. However, they also showed that the quantity of adipsin was increased in the absorbed placentas compared with the normal placentas, suggesting that local expression of adipsin has an effect at the maternal-fetal interface and probably plays a role in spontaneous abortion [104].

uNK cells have been suggested to have a critical function in pregnancy by promoting decidual health, appropriate vascularization of implantation sites, and placental size. In the murine pregnant uterus, extravillous cytotrophoblasts have invaded the maternal decidua. While the decidual macrophages or DCs recognize the trophoblast debris, uNK cells may become active and acquire a cytotoxic function like that of peripheral NK cells, propagating an immune attack on fetal organs and leading to abortion or premature fetal loss [105]. Recently, upregulation of Th17 cells and their related cytokines (e.g., IL-17 and IL-23) was observed in the blood and decidual tissues of patients with unexpected abortion [106]. Wang et al. also demonstrated that the suppressive activity of Tregs on Th17 cells was decreased in patients with unexplained recurrent miscarriages, that the ability of Tregs to repress inflammatory cytokine production may be effected by direct cell-cell contact, and that transforming growth factor-*β* and IL-10 could inhibit the expression of IL-17 [107]. Thus, it is likely that investigation of immunomodulatory molecules during pregnancy could assist in developing strategies for prevention or treatment of abortion or fetal loss. We herein focus on three immunoregulatory strategies: (1) induction of endogenous peroxisome proliferator-activated receptor-*γ* (PPAR*γ*) to reduce antioxidant stress and its related immunomodulation; (2) delivery of decoy receptor 3 (DcR3) to neutralize LIGHT (lymphotoxin exhibits inducible expression and competes with herpesvirus glycoprotein D for HVEM on T cells, LIGHT also known as TL4 or TNFSF14)/Fas signaling; (3) overexpression of galectin-9 to block the T-cell immunoglobulin mucin (TIM)-3 pathway and its potential immunomodulatory role in threatened pregnancy.

PPAR*γ* is a member of the nuclear receptor superfamily, a group of transcription factors that regulate expression of their target genes upon ligand binding. Endogenous ligands including oxidized fatty acids and prostanoids can bind to and activate the receptor [108]. Barak et al. reviewed the role of PPAR*γ* in the areas of adipocyte and macrophage biology, insulin action, bioenergetics, and inflammation and somewhat surprisingly found that PPAR*γ* plays an essential role in placental biology [109]. PPAR*γ* may also function in modulating fetal membrane signals toward parturition. In addition, Schaiff demonstrated the unique aspects of PPAR*γ* function in trophoblasts, which may have direct implications for the use of PPAR*γ* ligands during pregnancy [110]: PPAR*γ* agonists may decrease the risk of preterm delivery by suppressing the inflammatory response within the fetal membranes. Additional research that focuses on the mechanism of action, molecular targets, and functions of placental PPAR*γ* is paramount for the translation of these potentially beneficial functions of PPAR*γ* into therapeutic use during pregnancy [110]. Linoleic acid is a well-known component of many foods and is present in vegetables, fruits, nuts, grains, and seeds. Linoleic and linolenic acids are easily absorbed by oral intake to allow bioavailability to the plasma and the brain [111]. The conjugated form of linoleic acid, cis-9, trans-11, and a well-researched PPAR*γ* ligand was shown to be formed naturally from linoleic acid by gut flora, especially probiotics [112]. This suggests that appropriate nutrition, such as linoleic acids and linolenic acids combined with probiotics that are able to upregulate PPAR*γ* ligands, could provide benefits favoring an uncomplicated pregnancy.

TIM glycoproteins share common structural motifs, including a signal peptide, Ig domain, mucin domain, transmembrane domain, and intracellular tail, with phosphorylation sites [113]. TIM-3 was originally identified as a Th1-specific cell-surface molecule that downregulates Th1 responses through inducing apoptosis signaling by galectin-9 engagement [113, 114]. These results suggest that TIM-3 may modulate the Th1/Th2 balance. In addition, recent reports show that TIM-3 is also expressed on innate immune cells such as DCs and seems to promote innate immunity [115]. Such features of TIM-3 are consistent with the paradigm of Th1/Th2 shift and the activation of the innate immune system in pregnancy. Zhao et al. showed that in pregnant women, TIM-3 enhances both innate and adaptive immune responses by means of its upregulation in innate immune cells, and abnormalities of TIM-3 in pregnant woman may be deleterious to a normal pregnancy. Therefore, TIM-3 may be an indicator for predicting the risk of abortion in pregnant women [116]. In our recent study, we demonstrated that control of the pathogenic Th1 cell immune response through overexpression of galectin-9 to suppress TIM-3 signaling and downregulate proinflammatory cytokine production can inhibit the progressive destruction of *β* cells in autoimmune diabetes [117], a finding that may suggest a possible strategy for treatment of threatened pregnancy.

A soluble decoy receptor, DcR3, that binds to FasL and inhibits FasL-induced apoptosis has been identified [118], and FasL and LIGHT are established as ligands of DcR3 [119, 120]. Functionally, DcR3 can block FasL/LIGHT-mediated apoptosis leading to the escape of cells from immune attack. TNF-like ligand 1A (TL1A), the third ligand of DcR3, is a costimulator of T cells that promotes IL-2 responsiveness and increases secretion of proinflammatory cytokines both *in vitro* and *in vivo*. In addition, DcR3 suppresses TL1A-induced NF-*κ*B activation and apoptosis [121]. Of note, Gill and Hunt postulate that placental cytotrophoblast cells are protected from LIGHT-mediated apoptosis by both soluble receptor DcR3 and cellular inhibitors of apoptosis-2 to protect human cytotrophoblast cells against LIGHT-mediated apoptosis [122]. In addition, Yen et al. demonstrated that human gestational tissues showed differential production of DcR3, and that decidual DcR3 protein was lower in anembryonic than normal pregnancies [123]. We have shown the immunomodulatory and therapeutic activity of DcR3 in various experimental autoimmune disorders in nonobese diabetic mice [124, 125], experimental autoimmune experimental encephalomyelitis [126], and murine autoimmune crescentic glomerulonephritis [127], suggesting a potential activity of DcR3 in the regulation of successful pregnancies. However, above so-called potential immunomodulatory molecules are only the tips of icebergs in the understandings of the complex immunopathogenic mechanisms of threaten pregnancy. Nevertheless, further studies are essential to clarify these hidden mysteries.

## 9. Conclusion

The immunologic bond between mother and fetus remains a mystery, although current advances in molecular immunobiology have clarified many of the parameters involved in the fetomaternal interaction during implantation. Experimental models provide major insights in the field of reproductive immunology and the immunomodulation of normal or pathological pregnancy. However, ethical issues concerning the study of the physiology of early pregnancy in humans, together with the difficulty of generalizing animal findings to humans, are basic impediments to the clarification of the implantation process and its subsequent investigation [128].
